# Mass spectrometry-based metabolomics for the discovery of candidate markers of flavonoid and polyphenolic intake in adults

**DOI:** 10.1038/s41598-021-85190-w

**Published:** 2021-03-11

**Authors:** David Charles, Lee A. Gethings, James F. Potts, Peter G. J. Burney, Vanessa Garcia-Larsen

**Affiliations:** 1 Barts and the London Medical School, Garrod Building, Turner St, Whitechapel, London, UK; 2 Waters Corporation, Stamford Avenue, Wilmslow, SK9 4AX UK; 3 National Heart and Lung Institute, Imperial College London, London, UK; 4 Program in Human Nutrition, Department of International Nutrition, The Johns Hopkins Bloomberg School of Public Health, 615 N Wolfe St, Baltimore, MD 21205 USA

**Keywords:** Biological techniques, Biomarkers, Medical research

## Abstract

Robust biological markers of dietary exposure are essential in improving the understanding of the link between diet and health outcomes. Polyphenolic compounds, including flavonoids, have been proposed to mitigate the risk of chronic diseases where oxidative stress and inflammation play a central role. Biomarkers can provide objective measurement of the levels of polyphenolic compounds. In this study, we provide methodology to identify potential candidate markers of polyphenol intake in human serum. Seventeen participants from the UK arm of the Global Allergy and Asthma Network of Excellence (GA^2^LEN) had their dietary intake estimated using a validated food frequency questionnaire, and serum samples were assessed using mass spectrometry to identify potential candidate markers. 144 features were assigned identities, of these we identified four biologically relevant compounds (rhamnazin 3-rutinoside, 2-galloyl-1,4-galactarolactone methyl ester, 2″,32″-di-O-p-coumaroylafzelin and cyclocommunin), which were significantly increased in the serum of participants with high predicted level of fruit and vegetable intake. 2-galloyl-1,4-galactarolactone methyl ester was strongly correlated with total flavonoids (r = 0.62; *P* = 0.005), flavan-3-ols (r = 0.67; *P* = 0.002) as well as with other four subclasses. Rhamnazin 3-rutinoside showed strong correlation with pro-anthocyanidins (r = 0.68; *P* = 0.001), flavones (r = 0.62; *P* = 0.005). Our results suggest that serum profiling for these compounds might be an effective way of establishing the relative intake of flavonoids and could contribute to improve the accuracy of epidemiological methods to ascertain flavonoid intake.

## Introduction

Inflammation and oxidative stress are potentially major factors in the development of non-communicable chronic diseases^1,2^. Polyphenolic compounds are plant-derived compounds, widely found in fruits and vegetables. The two largest groups of polyphenolic compounds are phenolic acids, which include gallic acid and ellagic acid, and flavonoids, a family of compounds with two phenyl rings and a heterocyclic ring. Extensive experimental evidence shows that polyphenolic compounds can contribute to counteract inflammation, through several mechanisms, including the reduction of production of inflammatory cytokines, and to have antioxidant properties^3,4^. Increasing epidemiological evidence has demonstrated the potential benefits of these plant compounds in the prevention of non-communicable diseases (NCDs) such as cardiovascular (CVD)^5^, cancer, neuro-degenerative^6,7^, allergic, and respiratory diseases^3,8^.

Due to their potential role in the prevention of disease and the preservation of health, investigating consumption of dietary flavonoids is of major interest for epidemiologists. Population-based studies usually rely on self-reported dietary questionnaires, which collect data on intake of foods over a defined period, ranging from days (24-h recall questionnaires) to months (food frequency questionnaires [FFQs]). Although these instruments are helpful, and can provide reliable estimates of usual dietary intake, they have inherent methodological limitations that reduce their ability to accurately estimate food intake^9^. The standard method to derive flavonoid intake is to combine food intake estimates with food composition tables that contain variable sources and estimates of flavonoid^10^. Contents of these flavonoids vary between databases as these are greatly influenced by multiple factors such as food variety and chemical methods^11^.

Upon consumption, flavonoids are extensively metabolised in the gastrointestinal tract, the liver, and by the colonic microbial metabolism, in phase I and phase II metabolic pathways^12^. These processes are heavily influenced by a number of intra-individual factors^13,14^, making the identification of flavonoid-related compounds challenging.

The development of fast, accurate, and objective methods to estimate markers of dietary intake, including flavonoids, could enable improved understanding of the complex relationship between disease and dietary exposures. Such methods would help to reduce the inherent biases from data derived from dietary questionnaires, and reduce measurement error^15^. Metabolomics using mass spectrometry provides a comprehensive approach for identifying biologically relevant biomarkers of food intake. This study aims to identify potential candidate markers of polyphenolic compound intake in vivo, which could serve as a basis for further exploration in larger studies.

## Methods

### Participants and samples

Serum samples from 20 participants of the UK arm of GA^2^LEN (Global Asthma, and Allergy Network of Excellence) survey^16^ from two centres in London and Southampton, were used for this pilot investigation. The GA^2^LEN survey is a multinational European study that focuses on investigating environmental and genetic risk factors for asthma and poor lung function in European adults. Samples were obtained between 2004 and 2006 and were stored at -80 degrees Celsius until analysed. The 20 samples were chosen from the 10 participants who had the highest intakes of flavonoids and the ten participants with the lowest. The laboratory was blind to the eligibility criterion of the samples.

### Dietary assessment of flavonoid intake

As part of the GA^2^LEN Follow-up Survey, we designed an FFQ that could be used as a single, standardized instrument to ascertain dietary intake across countries, and to facilitate international comparisons^17^. Participants were asked for their average intake of 250 food items in the past 12 months. Daily estimates were derived converting portion sizes into grams. Total energy and nutrient intakes were estimated using the UK Food composition Table^18^. The USDA tables for flavonoid content of foods^19,20^ were used to derive estimates of the major subclasses of flavonoids: flavanones (eriodictyol, hesperetin, and naringenin), anthocyanins (cyanidin, delphinidin, malvidin, pelargonidin, petunidin, and peonidin), flavan-3-ols (catechins and epicatachin), flavonols (quercetin, kaempferol, myricetin, and isohamnetin), flavones (luteolin and apigenin), flavonoid polymers (proanthocyanidins, theaflavins and thearubigins). Total flavonoid intakes were derived by the addition of the six component subclasses. We also derived content of the pro-anthocyanidins subclass, which were derived separately, summing monomers, dimers, trimers, 4- to 6-mers, 7- to 10-mers, and > 10-mers.

### Mass spectrometry

#### Sample preparation

The participant’s sera were prepared for liquid chromatography/mass spectrometry (LC/MS) analysis as previously described^21^. Briefly, samples were thawed on ice for 60 min before aliquoting 400 µL into a 2.0 mL microcentrifuge tube containing 1.2 mL methanol and then mixed using a vortex mixer for 15 s, prior to centrifuging at room temperature for 15 min at 16,000 g. Samples were reconstituted using water with 0.1% formic acid prior to LC/MS analysis. A quality control (QC) sample based on a pool of all samples from both participant groups was also constructed (n = 20). Seventeen samples met QC, and were divided into 300 µL aliquots using 2.0 mL microcentrifuge tubes, and dried down overnight in a centrifugal vacuum evaporator with no additional heating applied.

#### Serum profiling by LC–MS

The participant’s sera were prepared for liquid chromatography/mass spectrometry (LC/MS) analysis as previously described^21^. Chromatographic separation of the samples was conducted with an ACQUITY I-Class system equipped with a 1.7-µm bridged ethylene hybrid (BEH), 100 mm × 2.1 mm C18 column (Waters Corporation, Milford, MA) and the column was maintained at 45ºC. Mobile phase A consisted of water with 0.1% formic acid and mobile phase B consisted of methanol with 0.1% formic acid. The analytes were resolved with a 16 min gradient from 0 to 100% mobile phase B at 400 µL/min. 3 µL of each sample was injected in triplicate on column and analysed in a randomised order. The pooled QC sample was injected every tenth injection. MS data were acquired in negative ion mode using a hybrid quadrupole-oaToF Synapt G2-Si QTof mass spectrometer (Waters Corporation, Wilmslow, United Kingdom) operated in resolution mode of analysis with a resolving power of 25,000 FWHM. Data were real-time lock mass corrected using the singly charged precursor ion of Leu-Enkephalin, which was acquired with a sampling frequency of 20 s. The capillary and cone voltages were 2.1 kV and 35 V, respectively.

Accurate mass data were collected in a data-independent acquisition (DIA) mode termed MS^E^ by alternating the energy applied to the collision cell between a low and elevated state. In low energy MS mode, data were collected at constant collision energy of 4 eV (per unit charge). In the elevated energy mode, the collision energy was ramped from 14 to 45 eV (per unit charge) during each integration. The spectral acquisition time in each mode was 0.18 s with a 0.02 s interscan delay. One cycle of low and elevated energy data was acquired every 0.4 s. The quadrupole mass analyser was operated in non-resolving mode and the LC-DIA-MS acquisition range from 50 to 2000 m/z^21^.

#### Data analysis

The metabolomics LC–MS data were aligned and normalized using Progenesis QI v2.0 (Nonlinear Dynamics, Newcastle upon Tyne, United Kingdom)^22^. Total ion current normalization was conducted using a two group experimental design with each group consisting of data from thirty (low dose)/thirty (high dose) (three technical replicates per ten (low dose)/ten (high dose) subject samples) LC-DIA-MS runs. The data were pre-processed using Progenesis QI. The resulting data matrix was imported into EZinfo v3.0 (Umetrics, Umeå, Sweden) for multivariate statistical analysis, using principal component analysis followed by orthogonal projection to latent structures discriminant results (OPLS-DA) to identify group differences based on covariance and correlation. Pareto scaling was used in which each variable was centred and multiplied by 1/√Sk, where Sk is the standard deviation of the variable. Identification of variable (metabolites) perturbations was achieved by examination of the OPLS-DA results where variables with high covariance and correlation where selected for identification. Compound identifications which result from the database search were then manually interrogated against the six main subclasses of flavonoids estimated from the FFQ. These metabolites were tentatively identified on the basis of accurate mass, isotopic fit and fragmentation (derived in-silico) using a combination of the Human Metabolome Database (http://www.hmdb.ca/) and LIPID MAPS (http://www.lipidmaps.org/). Resulting identifications were further curated based on a mass difference between observed and theoretical being < 5 ppm.

#### Identification of relevant flavonoid metabolites

Of the initial 42,287 features discovered in serum, 144 were manually assigned identities obtained from the Human Metabolome Database. Data were subsequently filtered to remove ions absent in at least 75% of the participants, enabling identification of 71 markers which allowed continuous measurement of flavonoid levels in vivo. We then manually identified metabolites that corresponded to any of the six main subclasses of flavonoids estimated from the FFQ. Based on the Metabolomics Standards Initiative^23^, compounds were annotated as level II (putatively identified compounds) and are summarized in Supplemental Table 1. Corresponding fragment ion spectra for the curated compounds are also provided as Supplemental Figures S1–S4.

**Table 1:** General characteristics and dietary intake in participants with low and high intakes of flavonoids.

Variables	Low intake group	High intake group	Total
Age in years (Mean (SD))	52.1 (12.6)	58.6 (10.2)	55.4 (11.6)
Male (N (%))	7 (70)	5 (50)	12 (60)
**Median (Interquartile range)**			
Total energy intake (kcal)	1501 (1224, 1954)	3066 (2537, 3341)	2284 (1257, 2891)
Total fruit intake (g)	34.7 (3.6, 55.7)	671.6 (444.6, 803.2)	353.1 (31.9, 571.8)
Total vegetable intake (g)	128.5 (77.4, 162.0)	422.1 (304.9, 494.1)	275.3 (91.7, 390.3)
Total flavonoids (mg)	32.7 (14.7, 49.4)	1047.4 (987.3, 1121.4)	540.1 (36.7,1027.4)
Flavanones (mg)	3.0 (0.1, 5.0)	64.2 (25.9, 45.5)	35.2 (1.6, 32.8)
Anthocyanins (mg)	2.4 (0.0, 4.5)	55.6 (40.8, 70.4)	30.4 (3.0, 61.8)
Flavan-3-ols (mg)	3.8 (2.9, 6.1)	182.2 (158.9, 225.9)	93.0 (3.6, 176.5)
Flavonols (mg)	5.9 (3.3, 9.5)	45.6 (40.8, 48.0)	25.7 (3.7, 45.3)
Flavones (mg)	0.5 (0.2, 0.7)	5.3 (3.1, 6.1)	2.9 (0.6, 5.5)
Polymers (mg)	17.6 (3.9, 27.6)	624.5 (641.4, 755.2)	356.1 (18.8, 708.9)
Pro-anthocyanidins (mg)	20.9 (5.7, 31.6)	454.0 (373.1, 534.4)	237.5 (23.0, 457.3)

### Statistical analysis

Predicted dietary intake of total flavonoids, flavonols, flavan-3-ols, flavones, flavanones, flavonoid polymers and anthocyanins was assessed against corresponding levels of identified molecules in the serum of patients using Spearman’s Correlation coefficient. A multivariate analysis in the form of unsupervised principal component analysis (PCA) was used to examine and evaluate structured variation in the dataset. Once group separation was established, orthogonal projections to latent structures-discriminant analysis (OPLS-DA) was employed to explain the variation with respect to the metabolomic data, whereby variables with high covariance and correlation were selected for compound identification. Statistical analyses were performed using STATA 12 (STATA StataCorp, College Station, TX, USA).

### Ethics

Written informed consent was obtained from all participants. The GA^2^LEN Follow-Up Survey was granted ethical approval by the UK’s National Ethics Research Committee (NERC) No. 07/H0604/121. All methods were carried out in accordance with relevant guidelines and regulations.

## Results

### Characteristics of participants

**Figure 1:**
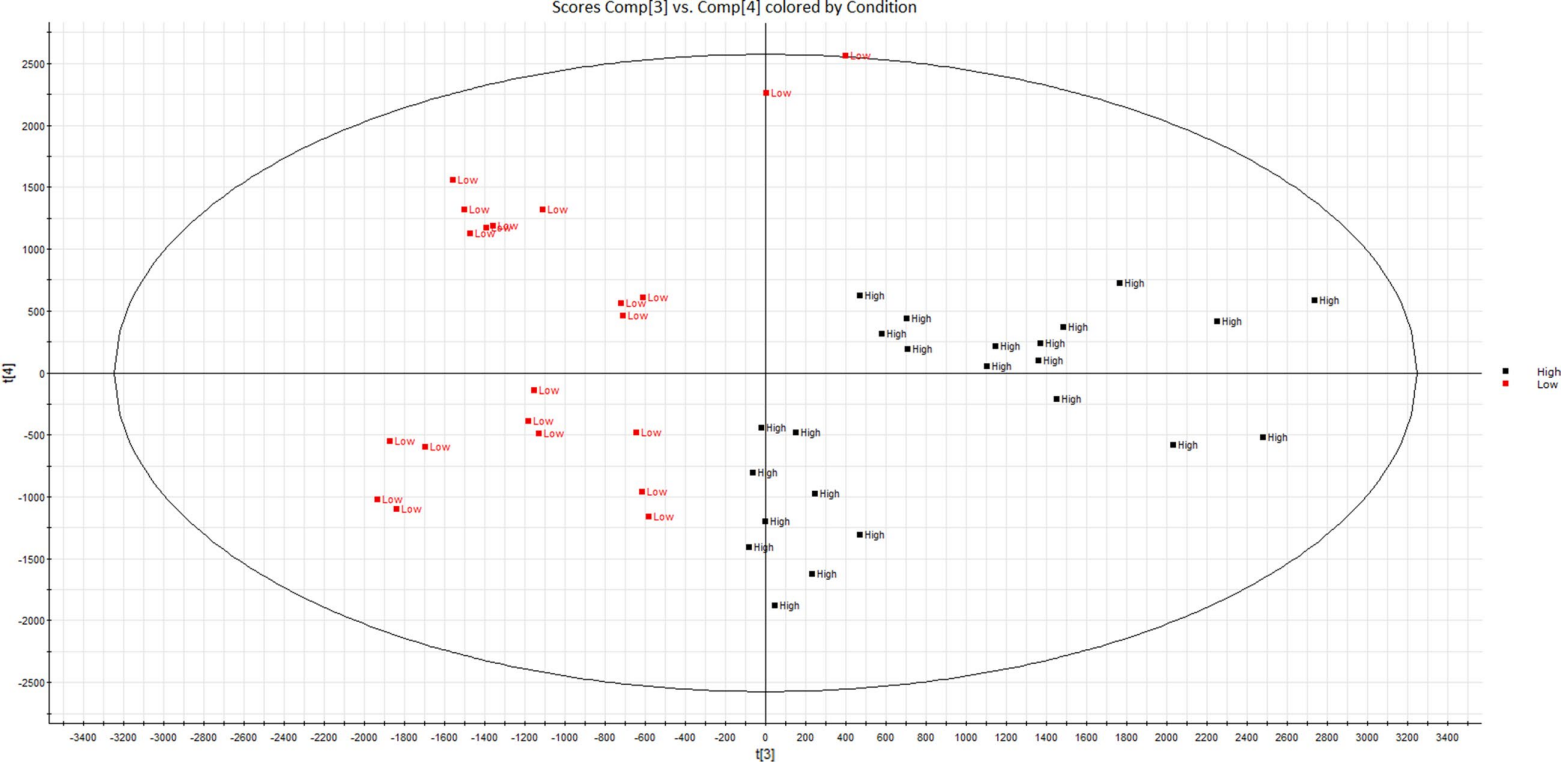
Unsupervised PCA (PC3 vs. PC4) scores plot illustrating separation between subjects of high (black) and low (red) total flavonoid intake.

**Figure 2:**
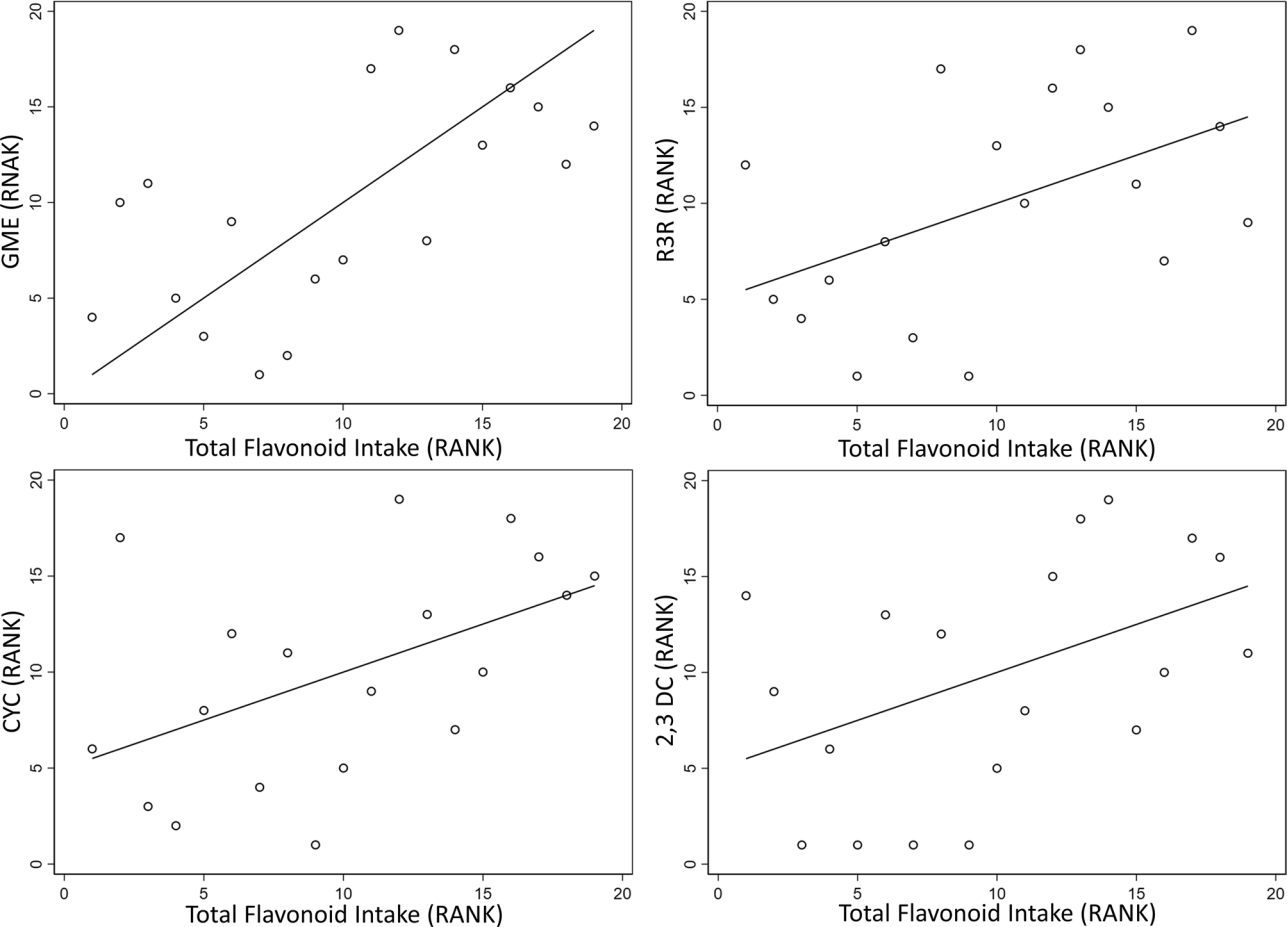
Scatter plot of predicted intake of flavonoids based upon FFQ compared measures of serum biomarkers.

Table 1 shows the general characteristics of the population used for this analysis. The mean age of participants was 55.4 years. Overall, median intake of fruits and vegetables was 353.1 g/day and 275.3 g/day, respectively. Total flavonoid intake reached a median of 540.0 mg/day, with intake of the polymer subclass being the highest subclass of flavonoid consumed. This was followed by flavon-3-ols, anthocyanins and flavonols. Figure 1 shows, through PCA, a clear separation of the low and high flavonoid intake groups as a result of metabolite differences between the two groups. Figure 2 shows the scatter plots of predicted intake of total flavonoids based upon FFQ compared measures of serum biomarkers.

### Characterisation of candidate markers in serum

Several candidate markers were found to be uniquely elevated in participants with high dietary intake of fruits and vegetables. These compounds were identified as being derivatives of common polyphenolic nutrients including flavonoids. Four compounds with a polyphenolic structure were found to be high in individuals with higher intakes of flavonoids, namely rhamnazin 3-rutinoside, 2″,3″-di-O-p-coumaroylafzelin, cyclocommunin (Brosimone I) and 2-galloyl-1,4-galactarolactone methyl ester. Following multi-variate statistical analysis to identify markers of significance, the database searching which subsequently followed for those markers did not provide any identifications related to polyphenol metabolites based on the search criteria referred to in the methods section.

### Potential markers associated with total flavonoid Intake

Serum compounds were compared against total dietary flavonoid intake. Several key compounds showed a good correlation with total flavonoid intake (Table 2). 2-galloyl-1,4-galactarolactone methyl ester (r = 0.62, *P* value = 0.005) showed the highest correlation with total flavonoid intake, followed by cyclocommunin (r = 0.50, *P* value = 0.03), and by rhamnazin 3-rutinoside (r = 0.50, *P* value = 0.03).

**Table 2:** Spearman’s correlation coefficient (rho) between dietary intake of flavonoids estimated from an FFQ with serum levels* of flavonoid metabolites (n = 17).

Subclasses of flavonoids estimated from dietary intake	2G1,4GME		R-3-R		CMN		2,3DC	
	rho	*P* value	rho	*P* value	rho	*P* value	rho	*P* value
Total flavonoids	0.62	**0.005**	0.50	**0.03**	0.50	**0.03**	0.46	0.05
Flavan-3-ols	0.67	**0.002**	0.46	0.05	0.36	0.13	0.40	0.09
Flavonols	0.63	**0.004**	0.51	**0.03**	0.33	0.17	0.27	0.26
Flavones	0.59	**0.015**	0.62	**0.005**	0.50	**0.03**	0.43	0.07
Polymers	0.62	**0.004**	0.64	**0.003**	0.45	**0.05**	0.46	0.05
Pro-anthocyanidins	0.55	**0.02**	0.68	**0.001**	0.47	**0.04**	0.56	**0.01**

Values in bold indicate a statistically significant *P* value (<0.05).

*Based on specific metabolite abundance in serum; 2G1,4GM—2-Galloyl-1,4-galactarolactone methyl ester; R3R—Rhamnazin 3-rutinoside; CMN—Cyclocommunin; 2,3DC—22″,32″-Di-O-p-coumaroylafzelin.

### Candidate biomarkers associated with flavonoid sub-classes

The metabolite rhamnazin 3-rutinoside (R-3-R), was highly correlated with intake of flavonols (r = 0.51, *P* value = 0.03). Rhamnazin 3-rutinoside also showed positive correlation with several related molecules including flavones (r = 0.62, *P* value = 0.005), pro-anthocyanidins (r = 0.68, *P* value = 0.001) and polymers (r = 0.64, *P* value = 0.003). The pyrano-flavonoid cyclocommunin was found to be most strongly associated with pro-anthocyanidins (r = 0.56, *P* value = 0.01).

Other polyphenolic compounds raised in individuals with high predicted flavonoid intake were also shown to be highly correlated with flavonoid sub-classes. 2-galloyl-1,4-galactarolactone methyl ester, which is related to gallic acid, was also shown to be highly correlated with estimated intake of flavonol (r = 0.62, *P* value = 0.005). Additionally, 2-galloyl-1,4-galactarolactone methyl ester was positively associated with all flavonoid subclasses, the highest association being with flavan-3-ols (r = 0.67, *P* Value = 0.002).

## Discussion

The objective of this study was to identify potential candidate markers of polyphenolic intake and to further elucidate the metabolic profiles of adults with high dietary intake of fruits and vegetables. This exploratory profiling identified four marker candidates, namely rhamnazin 3-rutinoside, 2-galloyl-1,4-galactarolactone methyl ester, 22″,32″-di-O-p-coumaroylafzelin and cyclocommunin, which were raised in individuals with high source-food intake.

Upon consumption, dietary flavonoids are extensively metabolized by phase I and phase II metabolism (which takes place predominantly in the gastrointestinal tract and liver) and colonic microbial metabolism^12^. A number of factors inherent to the individual will further affect these metabolic pathways. Intra-individual variations of flavonoid metabolism are likely to be present regardless of how homogeneous the sample might appear. As recently observed^13^, there are multiple intra- and inter-individual factors that can affect flavonoid metabolism after consumption, including age, sex, and genotype. These characteristics make the assessment of markers of flavonoid intake particularly challenging compared to the assessment of other biomarkers of dietary intake.

Nutritional metabolomics is an important developing field, which aims to discover new biomarkers of nutritional exposure and to help validate dietary assessment measures. Previous studies have identified sensitive biomarkers of specific fruits by profiling samples from groups of low and high consumers of specific target foods^24^. However, due to the lack of biomarkers of polyphenols, epidemiological studies still heavily rely on dietary questionnaires to ascertain usual dietary intake. There is some evidence supporting the use of metabolomics to discriminate high and low intakes of epigallocatechin-3-gallate (EGCG) and epicatechin-3-gallate (ECG), in individuals who have regularly consumed green tea over a month, and plasma concentrations of genistein and daidzein have been identified as potential markers of intake of isoflavonoids^25^. However, there is very little evidence of possible candidate markers of intake of common subclasses of flavonoids^26^.

One of the potential markers we identified is 2-Galloyl-1,4-galactarolactone methyl ester. This compound has been detected in fruits, (https://hmdb.ca/metabolites/HMDB0037200). Therefore, its measurement in serum could be used as a potential marker for the consumption of these foods. Rhamnazin 3-rutinoside was strongly correlated with pro-anthocyanidins, and with its main group, polymers, and it could potentially be used as a marker of this subclass of flavonoid. Pro-anthocyanidins are being increasingly recognized for their potential protective effect against general mortality^27^, cardiovascular disease^28^, and for preserving lung function^8^. However, our findings of the absorption of glycosylated polyphenols/flavonoids are unexpected, as these usually undergo some phase II metabolism^29^.

Because of the extensive metabolisation of flavonoids after consumption, it is difficult to capture more ‘intact’ molecules in the bloodstream. Studies measuring flavonoids in plasma have usually relied on short-term intake of specific food sources of the flavonoid under study^26^. This is not very practical in population-based studies, where usual consumption over a prolonged period is ascertained to investigate the association of flavonoid and health outcomes.

To our knowledge, this is the first exploratory study to identify several potential candidate markers of flavonoid intake using sera from adults. We used a validated FFQ specifically designed to ascertain usual intake of fruit and vegetables over the past year in the studied population^17^, and included a wide range of foods rich in polyphenolic compounds. The PCA clearly discriminated the subjects with higher and lower intakes and the compounds identified in serum.

There are limitations in the present study. Although, we used a well established metabolomic strategy for identifying candidate biomarkers, the results of the study should be considered preliminary meriting further validation within the context of larger studies. The small sample studied prevented us from making meaningful assumptions about the possible influence of physical activity, body weight, or consumption of other foods that could alter the results. Finally, although the FFQ that we used had been previously validated, all dietary instruments have inherent limitations related to recall bias and measurement error, particularly when reporting intake of foods that are perceived as healthy.

## Conclusion

In this study we demonstrate that four potential candidate biomarkers rhamnazin 3-rutinoside, 2-galloyl-1,4-galactarolactone methyl ester, 2″,3″-di-O-p-coumaroylafzelin and cyclocommunin are raised in individuals with high intakes of fruits and vegetables and that quantities of these biomarkers in the sera are strongly correlated with predicted flavonoid intakes. Further work to optimise and utilise these methods might enable more rapid characterisation of a population’s flavonoid fingerprint.

## Supplementary Information

Supplementary Information 1.

Supplementary Information 2.

Supplementary Information 3.

Supplementary Information 4.

Supplementary Information 5.
