# Supplementary material for: Mass spectrometry-based metabolomics for the discovery of candidate markers of flavonoid and polyphenolic intake in adults
Source: Sci Rep. 2021 Mar 11;11:5801. doi: 10.1038/s41598-021-85190-w (PMC7952705; doi:10.1038/s41598-021-85190-w)
Supplement: Supplementary file 1 — Supplementary Information 1. [file 41598_2021_85190_MOESM1_ESM.docx]

**Mass spectrometry-based metabolomics for the discovery of candidate markers of flavonoid and polyphenolic intake in adults**

David Charles^1^, Lee A. Gethings^2^, James F Potts^3^, Peter GJ Burney^3^, Vanessa Garcia-Larsen^4^*

1Barts and the London Medical School, Garrod Building, Turner St, Whitechapel, London, UK

2Waters Corporation, Stamford Avenue, Wilmslow, SK9 4AX, United Kingdom

3National Heart and Lung Institute, Imperial College London, UK

4Program in Human Nutrition, Department of International Nutrition, The Johns Hopkins Bloomberg School of Public Health, Baltimore, US

*Corresponding author:

Dr Vanessa Garcia-Larsen

Program in Human Nutrition, Department of International Nutrition,

The Johns Hopkins Bloomberg School of Public Health,

615 N Wolfe St, Baltimore MD 21201, US

E-mail: [vgla@jhu.edu](mailto:vgla@jhu.edu)

Supplementary Table 1 – Annotated MS/MS spectra for the compounds of interest

| **Compound Feature** | **Compound ID** | **Description** | **m/z** | **Retention time (min)** | **Adducts** | **Formula** | **Score** | **Fragment ion m/z** |
| --- | --- | --- | --- | --- | --- | --- | --- | --- |
| 0.75_357.0456m/z | HMDB0037200 | 2-Galloyl-1,4-galactarolactone methyl ester | 357.046 | 0.75 | M-H | C14H14O11 | 43.2 | 149.0455 |
|  |  |  |  |  |  |  |  | 188.0326 |
|  |  |  |  |  |  |  |  | 210.0169 |
|  |  |  |  |  |  |  |  | 325.0201 |
| 11.23_637.1763m/z | HMDB0037467 | Rhamnazin 3-rutinoside | 637.176 | 11.23 | M-H | C29H34O16 | 35.6 | 160.0741 |
|  |  |  |  |  |  |  |  | 519.1144 |
| 11.21_705.1634m/z | HMDB0039928 | 2'',3''-Di-O-p-coumaroylafzelin | 705.163 | 11.21 | M-H2O-H | C39H32O14 | 37.7 | 632.1535 |
|  |  |  |  |  |  |  |  | 673.1352 |
|  |  |  |  |  |  |  |  | 679.1457 |
|  |  |  |  |  |  |  |  | 687.1508 |
|  |  |  |  |  |  |  |  | 689.1665 |
| 3.99_839.3102m/z | HMDB0040480 | Cyclocommunin | 839.31 | 3.99 | 2M-H | C25H24O6 | 38.1 | 74.0162 |
|  |  |  |  |  |  |  |  | 159.0815 |
|  |  |  |  |  |  |  |  | 183.0815 |
|  |  |  |  |  |  |  |  | 203.0713 |
|  |  |  |  |  |  |  |  | 225.0557 |
|  |  |  |  |  |  |  |  | 314.0584 |
|  |  |  |  |  |  |  |  | 319.0611 |
|  |  |  |  |  |  |  |  | 731.2862 |

Legend to supplementary Figures S1-S4

Figure S1 MS/MS spectra for 2-galloyl-1,4-galactarolactone methyl ester

Figure S2 MS/MS spectra for 2”,3”-di-O-p-coumaroylafzelin

Figure S3 MS/MS spectra for cyclocommunin

Figure S4 MS/MS spectra for rhamnazin 3-rutinoside
