# Supplementary figures and images for: Mass spectrometry-based metabolomics for the discovery of candidate markers of flavonoid and polyphenolic intake in adults
Source: Sci Rep. 2021 Mar 11;11:5801. doi: 10.1038/s41598-021-85190-w (PMC7952705; doi:10.1038/s41598-021-85190-w)

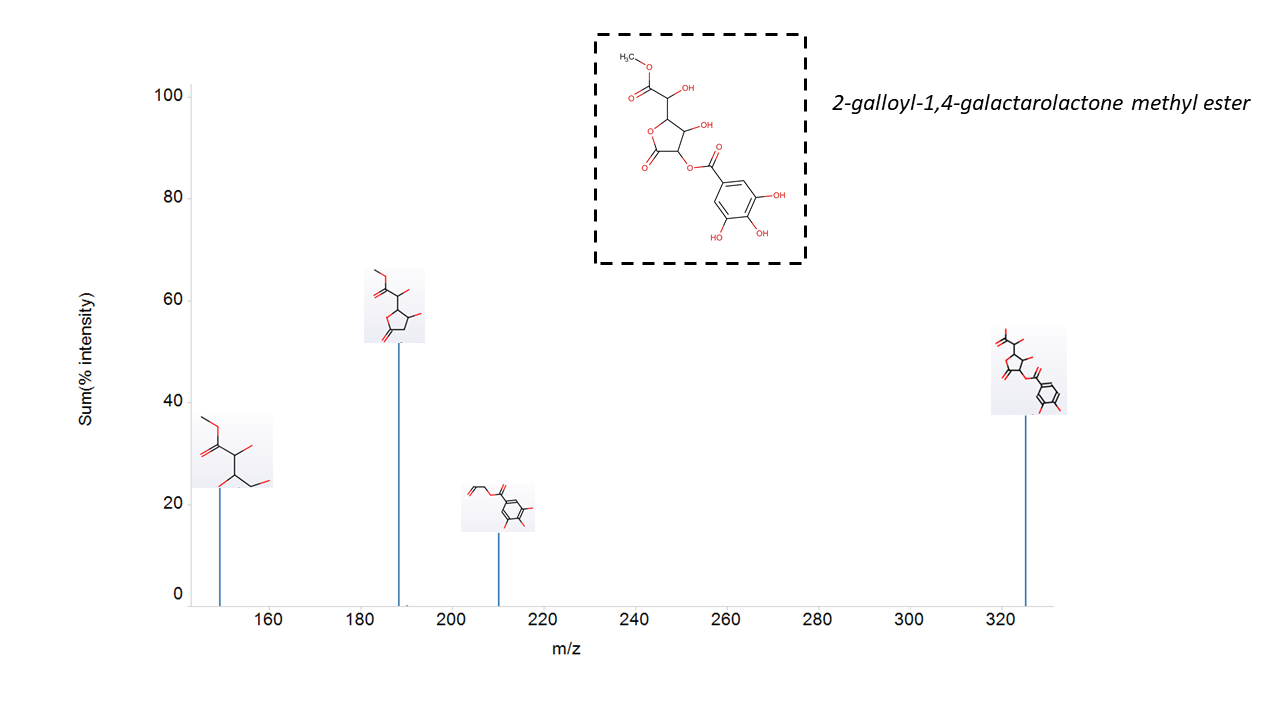

Supplement: Supplementary file 2 — Supplementary Information 2. [file 41598_2021_85190_MOESM2_ESM.tif]

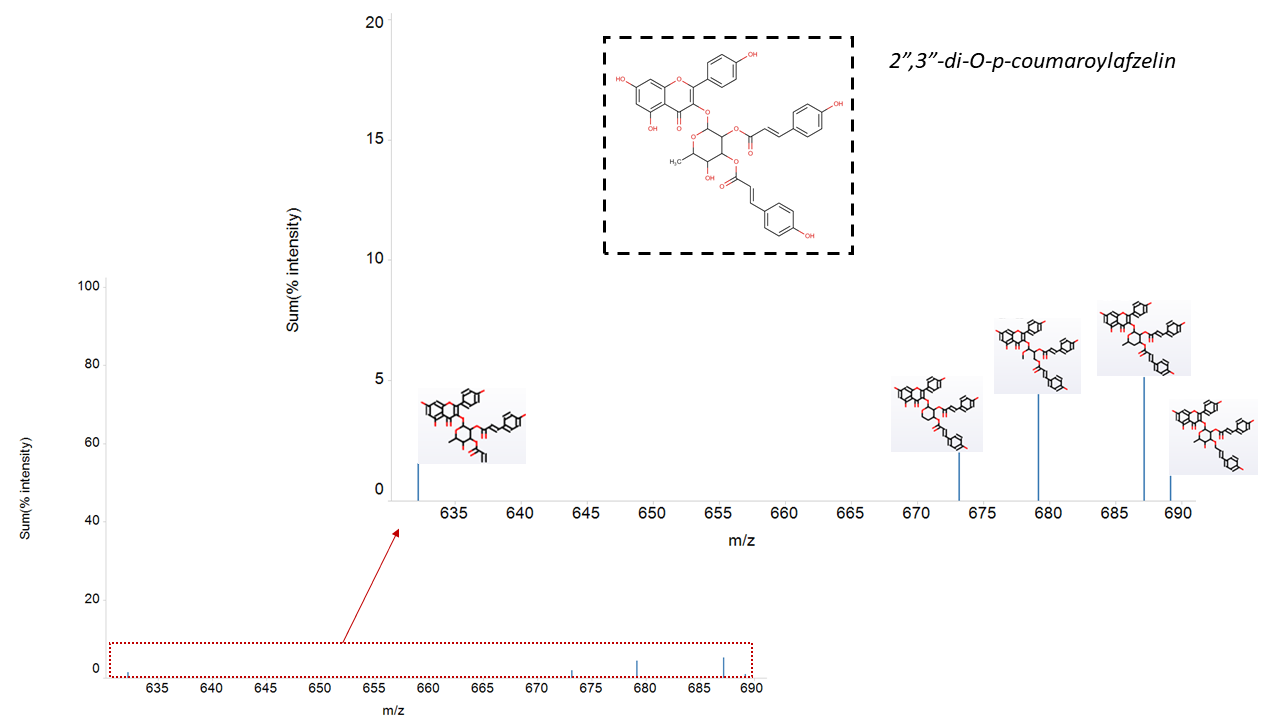

Supplement: Supplementary file 3 — Supplementary Information 3. [file 41598_2021_85190_MOESM3_ESM.tif]

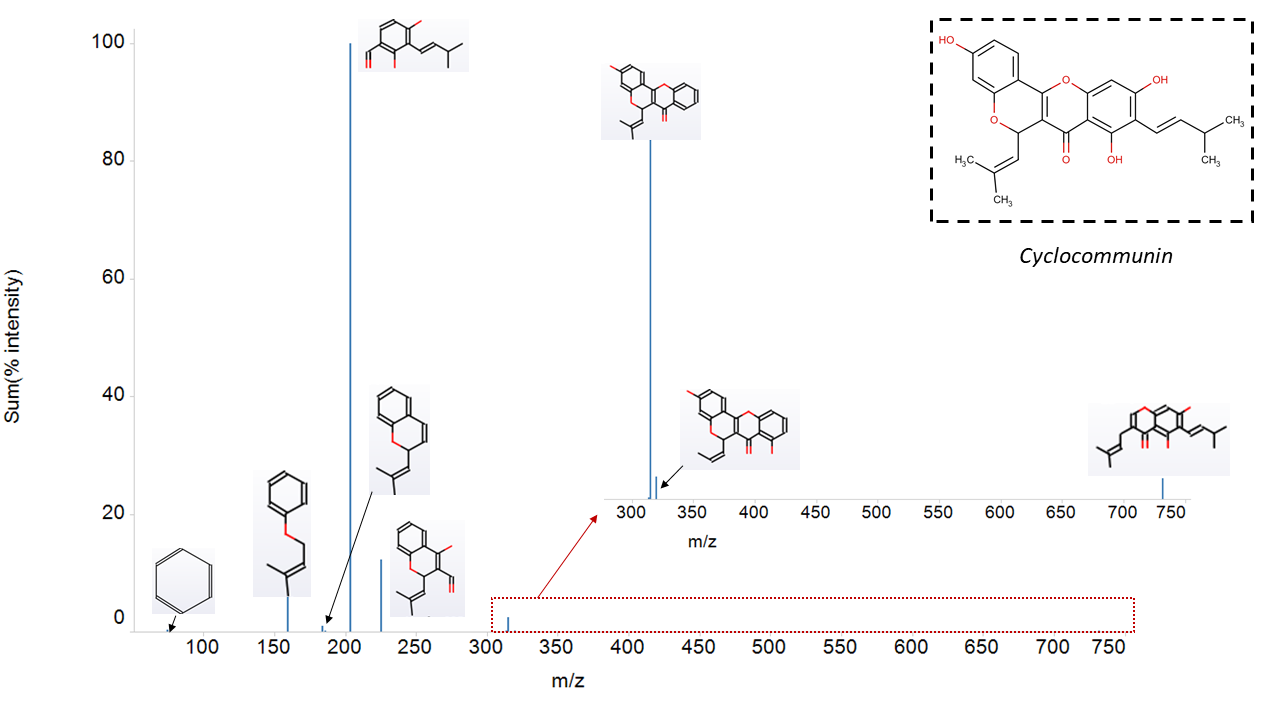

Supplement: Supplementary file 4 — Supplementary Information 4. [file 41598_2021_85190_MOESM4_ESM.tif]

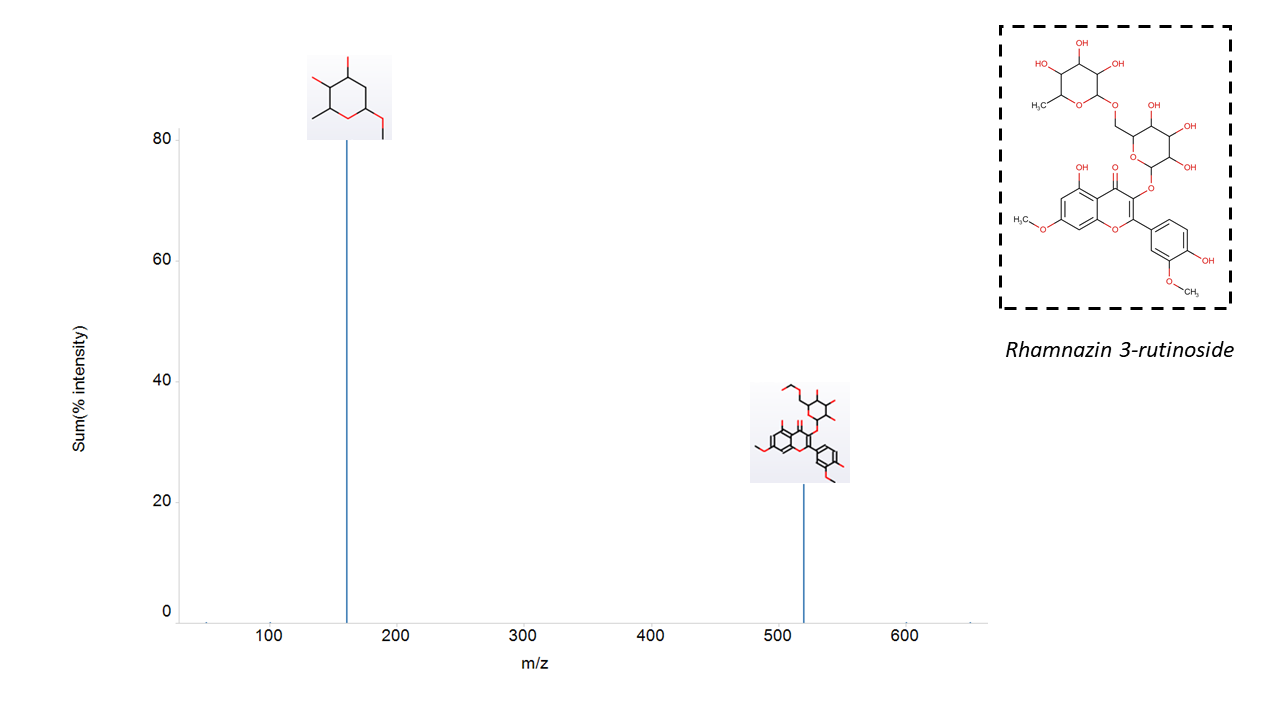

Supplement: Supplementary file 5 — Supplementary Information 5. [file 41598_2021_85190_MOESM5_ESM.tif]
